# The Resonance and the Allium ureteral stents in the treatment of non-malignant refractory ureterostenosis

**DOI:** 10.1186/s12894-021-00815-6

**Published:** 2021-04-07

**Authors:** Wei Gao, Tianying Xing, Tongwen Ou

**Affiliations:** Department of Urology, Xuanwu Hospital, Capital Medical University, Beijing, 100053 China

**Keywords:** Ureterostenosis, Retroperitoneal fibrosis, Resonance stent, Allium stent, Efficacy, Safety

## Abstract

**Background:**

Refractory non-malignant ureterostenosis is challenging to treat. The experience to treat the stenosis primarily cause by retroperitoneal fibrosis with the Resonance and Allium metallic stent is still limited. We aim to evaluate the efficacy and safety of these two stents and provide alternative treatment options.

**Methods:**

A retrospective study was conducted for patients with non-malignant ureterostenosis and treated with the Resonance and Allium stents from March 2011 to September 2020 in our department. The efficacy was evaluated by the change of serum creatinine, glomerular filtration rate (GFR), the proportion of GFR of the affected side and hydronephrosis grade. The safety was evaluated by postoperative presence of moderate or severe overactive bladder (OAB), recurrent urinary infection, pain, stent displacement, encrustation and re-obstruction.

**Results:**

33 patients were eligible for the study, including 18 cases treated by the Resonance stents and 15 patients treated by the Allium stents. The patients of two groups had similar age and gender proportion. The cause of ureterostenosis was mainly retroperitoneal fibrosis in both groups but the Resonance group had more idiopathic cases. Follow-up time was significantly longer in the Resonance group than the Allium group (36.2 ± 24.0 vs 9.4 ± 5.0 months, *p* < 0.001). Both groups showed improvement or maintenance of serum creatinine level, GFR, the GFR proportion of the affected side and hydronephrosis grade after treatment. The Resonance group presented significant higher incidence of moderate or severe OAB, recurrent urinary infection and pain, while the Allium group showed significant more cases of re-obstruction.

**Conclusion:**

Both the Resonance and Allium stent can relieve the non-malignant refractory ureterostenosis effectively. The Resonance stent may cause more irritable symptoms while the Allium stent may have a higher rate of re-obstruction. The long term efficacy and safety of the Allium stent in treating non-malignant refractory ureterostenosis requires further study.

**Supplementary Information:**

The online version contains supplementary material available at 10.1186/s12894-021-00815-6.

## Introduction

A ureteral stent can drain the upper urinary tract and preserve kidney function, thereby providing time for further treatment and improving the patient’s quality of life. However, the conventional plastic or polymeric stents may become occluded after several months and require frequent change. Importantly, a typical plastic or polymeric ureteral stent often fail to relieve the hydronephrosis and provide insufficient resistance to the traction displacement of the ureter secondary to some refractory conditions, such as primary or metastatic retroperitoneal malignancies [1]. Thus, metallic ureteral stents are widely used.

Currently, there are two common types of commercial metallic stents: the Resonance Metallic Ureteral Stent and Allium Ureteral Stent. The Resonance stent has potent tensile strength and provides good drainage. In addition, it is resistant to extrinsic compression and occlusion. These properties allow longer dwelling time and less frequent stent exchanges. Thus, this stent provided a long-term renal protective effect and might be more cost-effective [2]. Allium stent is a self-expanding stent made of a super elastic nitinol skeleton and with biocompatible polymeric cover. The metal component provides radial and longitudinal strength, while the polymer bio-inertness prevents tissue ingrowth into the lumen and early encrustation. Both two stents have shown good efficacy in treating malignant ureteral strictures [3, 4].

Several benign disorders may cause refractory ureteral obstruction, including idiopathic retroperitoneal fibrosis (IRPF) and secondary retroperitoneal fibrosis (SRPF), iatrogenic injury during ureteral lithotripsy. Retroperitoneal fibrosis is rare and characterized by extensive fibrosis and inflammation in the retroperitoneal region, frequently causing extrinsic compression of the ureter. Ureterolysis and nephrostomy are the treatment options, but they may be time-consuming, risky and impact quality of life [5, 6]; less invasive but effective treatment ways are in urgent need. Meanwhile, traditional stents may fail to relieve the obstruction because of insufficient strength. Although metallic stents are suitable for malignant obstruction, their efficacy and safety for the treatment of non-malignant ureteral strictures still needs further evaluation. Thus, we reviewed patients with non-malignant ureterostenosis (primarily retroperitoneal fibrosis) treated by Resonance and Allium, and compared the efficacy and safety of the two different groups.

## Methods

### Study design

A retrospective study was conducted in patients with non-malignant cause of ureterostenosis treated by Resonance or Allium stent from March 2011 to September 2020 at Department of Urology, Xuanwu Hospital, Capital Medical University, Beijing, PR China. Inclusion criteria included 1. patients underwent traditional stents placement due to non-malignant ureteral stenosis but still had aggravated hydronephrosis or worse renal function; 2. Resonance (COOK, Bloomington, IN, USA) or Allium (Allium, Caesarea, Israel) stent was placed; 3. the placement of stents was performed by a single surgeon (Gao). Since radiotherapy in the corresponding area may cause urethral stricture in patients with history of malignancy, and the stricture is caused by SRPF secondary to radiation but not the malignancy itself, these patients were also included in the study if they have no evidence of tumor recurrence. Exclusion criteria included 1. patients with malignant tumors history who had radiation-caused SRPF ureteral stricture but showed evidence of recurrence or required further chemotherapy or radiotherapy; 2. patients with nephrostomy; 3. intraoperative placement failure and subsequent replacement with a different type of stent. This research was approved by the Ethics Committee of Xuanwu Hospital, Capital Medical University and conducted in accordance with the declaration of Helsinki. Each patient provided informed consent.

### Clinical practice

Before each operation, the patient underwent routine preoperative evaluation, including urine analysis, serum creatinine, abdominal Computed Tomography (CT) scan to evaluate hydronephrosis and renography (using Tc-99m-DTPA as the tracer) to evaluate glomerular filtration rate (GFR). Intraoperative placement of ureteral stents was usually under fluoroscopic guidance. For Resonance stents, 24 cm ones were chosen for patients shorter than 165 cm, 26 cm for patients between 165 and 175 cm, and 28 cm for patients taller than 175 cm, mostly referring to the previous literature but adjust for Asian population [7]. For Allium stents, the length and perimeter of the stent were selected according to the length and degree of the stenosis assessed by imaging. The availability of Allium stent was much later than the Resonance stent. When both stents were available and alternative, the use of the stent was mainly according to the patient’s selection after considering the potential benefit, side effects and cost. On the first day after surgery, X-rays were used in the postoperative settings to confirm placement. Follow-up was conducted postoperatively at 1 month, 3 month and 6 months and 1 year (with time range due to patients’ compliance), and the patient underwent urine analysis, blood creatinine, abdominal CT scan and renogram. For patients with good response and tolerance, the Resonance stents were replaced yearly.

### Efficacy and safety evaluation

For efficacy evaluation, blood creatinine, GFR and hydronephrosis grade at last follow up were compared with their preoperative values. Hydronephrosis grade was divided into 5 grades (0–IV) according to the CT images [8]. For safety evaluation, presence of recurrent urinary tract infection, moderate or severe overactive bladder symptom and flank pain were documented. Recurrent urinary infection was defined as 2 times urinary infection in the past 6 months and 3 times in the past year. Moderate or severe overactive bladder symptom was evaluated by Overactive Bladder Symptom Score (OABSS) with score equal or greater than 6. Also, complications discovered by CT images was recorded, including stent displacement, encrustation around the stent and re-obstruction.

### Statistical analysis

Continuous variables were described as mean ± standard deviation, and categorical variables was described as frequency or median. For patients’ demographics, the difference between the Resonance and Allium groups were examined by student *t* test for continuous variables, and Fisher’s exact test or Mann–Whitney U test were used for categorical variables. Paired t test or Mann–Whitney U test were used to examine the changes of efficacy parameters before and after treatment in each group. Fisher’s exact test was used to compare safety parameters. All tests were two-sided. *P* value < 0.05 was regarded as statistically different. SPSS 22.0. software was used for all statistical analysis.

## Results

### Patient demographics

We reviewed 128 patients who were treated with metallic ureteral stents for ureteral strictures during the study period. 33 of them met the inclusion criteria. Patient demographics were summarized in Table 1. Among the patients, 18 cases received the Resonance stents, and 15 patients received the Allium stents. The mean age and gender proportion of the two groups did not show significant difference. The treatment duration was 36.2 ± 24.0 months for the Resonance group, which was significantly longer than 9.4 ± 5.0 months for the Allium group. The cause of ureterostenosis included IRPF, SRPF (after radiotherapy) and ureteroscope lithotripsy. But the proportion of each cause was significantly different between the two groups: the Resonance group had more IRPF patients, while obstruction secondary to ureteroscope lithotripsy only existed in the Allium group.

**Table 1:** Patients demographics

	Resonance stent	Allium stent	*P* value
Number of patients	18	15	
Age (years)	55.7 ± 13.4	52.5 ± 12.5	0.487
Gender			
Male	12	9	0.731
Female	6	6	
Follow-up time (months)	36.2 ± 24.0	9.4 ± 5.0	< 0.001
Causes of stenosis			
IRPF	14	5	0.013
SRPF	4	6	
Ureteroscope lithotripsy	0	4	

*IRPF* idiopathic retroperitoneal fibrosis, *SRPF* secondary retroperitoneal fibrosis

### Efficacy and safety

Efficacy was evaluated by comparison of serum creatinine, GFR, GFR proportion of the affected side and hydronephrosis grade before operation and at last follow-up (Table 2). Both groups showed improved creatinine level and GFR, although they did not reach significant difference. The GFR proportion of the affected side increased in the Resonance group, but dropped minimally in the Allium group. The level of hydronephrosis relieved significantly in both groups. In general, Both the Resonance and the Allium group showed good efficacy to protect renal function and relieve hydronephrosis. However, Allium stents showed short term good efficacy as the follow-up time was significantly shorter in the Allium group.

**Table 2:** Efficacy evaluation of the Resonance and Allium stent

	Resonance stent			Allium stent		
	Preoperative	Postoperative	*P* value	Preoperative	Postoperative	*P* value
Serum creatinine (μmol/L)	213.7 ± 268.8	89.8 ± 22.3	0.065	96.3 ± 52.8	84.3 ± 27.6	0.349
GFR (ml/min)	28.4 ± 15.1	30.4 ± 13.7	0.422	33.0 ± 15.8	35.3 ± 15.7	0.414
Affected side GFR proportion^a^ (%)	49.0 ± 24.5	50.5 ± 25.6	0.455	44.7 ± 16.1	44.1 ± 17.1	0.693
Hydronephrosis grade (median)	3	2	0.004	2	1	0.001

^a^Affected side GFR proportion calculated as affected side GFR/affected side GFR + contralateral GFR

For the safety parameter, regarding to the patients’ complaint, the Resonance group showed a higher frequency of the presence of moderate to severe OAB, recurrent urinary infection and pain than the Allium group (all *p* < 0.05). Via CT scan, the Allium group revealed significant higher rate of re-obstruction and more cases of displacement. However, encrustation was more common in the Resonance group, but did not show significant difference (Table 3).

**Table 3:** Safety evaluation of the Resonance and Allium stent

	Resonance stent	Allium stent	*P* values
Moderate to severe OAB	7	0	0.009
Recurrent urinary tract infection	7	1	0.046
Pain	7	1	0.038
Displacement	1	4	0.152
Encrustation	11	1	0.255
Reobstruction	0	5	0.013

*OAB* overactive bladder

## Discussion

Compared with the ureteral obstruction caused by extrinsic compression of malignancies, non-malignant refractory ureterostenosis is rarer. It is challenging for urologists not only because of deteriorating renal function but also difficulty in choosing the appropriate treatment plan for the individual. Traditional silicone or plastic ureteral stents are most commonly used and cost-effective. Despite their advantages, they have shown a relatively high failure rate when used to treat some chronic ureteral stenosis, especially in patients with retroperitoneal fibrosis or iatrogenic injury. In our study, we reviewed patients treated by the Resonance or the Allium metallic stents for ureterostenosis caused by non-malignant reasons (primarily retroperitoneal fibrosis). Both two types of stents showed good efficacy to protect renal function from deterioration. The Resonance stents caused more irritative complications while the Allium stent had higher frequency of displacement and re-obstruction.

The majority of our cases were retroperitoneal fibrosis, including 18 IRPF and 11 SRPF. IRPF is related to autoimmune diseases and characterized by chronic non-specific inflammation of the tissues around the retroperitoneal aorta with progressive hyperplasia of fibrous tissue. The factors of SRPF include drug side effects, infection, trauma, dissection, radiation therapy, malignant tumors [9–12]. Fibrous tissue surrounds and compresses its adjacent structure with the ureters involved especially. In our study, IRPF cases were diagnosed by rheumatologists, and SRPF cases were caused by radiotherapy. Although SRPF patients had history of cancer, most of them underwent 18F-FDG-Positron Emission Tomography to rule out malignant ureteral obstruction, reinforcing the accuracy of SRPF diagnosis [13]. Although percutaneous nephrostomy or ureterolysis can drain the upper urinary tract and relieve the stenosis, it reduces the patients’ quality of life or confronts great operative difficulty. Metallic stents become promising ways to treat RPF in some refractory cases.

The Resonance metal stent has a special coil structure and alloy composition, making it resistant to external compression and the ingrowth of proliferative tissue. The usage of Resonance metal stents has been reported in patients with benign ureteral stenosis primarily due to ureteropelvic junction obstruction or benign stricture, showing good drainage and good tolerance in most of the patients [14–17]. Benson et al. reported no stent failure in 15 patients with benign obstruction after a median follow-up time of 14 months. López-Huertas et al. [16] reported that the Resonance stents succeeded in 92% of the patients with benign obstruction. Both of these studies included 1 case of IRPF without stent failure. The Allium stent is a self-expanding, large-caliber ureteral stent to treat benign or malignant ureteral stenosis. It displayed good efficacy in some benign conditions like post ureteroscope lithotripsy stricture. Moskovitz used Allium stents to treat benign urinary stricture after endoscopic lithotripsy, urinary diversion and renal transplant. All stents were patent except from 1 case of endoscopic ureteral lithotripsy after 11 months [18]. The data regarding to RPF was still lacking. Our study compared the efficacy of the Resonance and the Allium stents in benign cases, primarily RPF. The result was favorable in protecting the renal function and reliving the hydronephrosis. This was expectable as both the two stents had greater tensile strength than traditional stents. Although the creatinine level and GFR did not increased significantly, both stents could improve the renal function or protect renal function from deteriorating. However, besides the successful cases in our study, we also experience 2 intraoperative failure when placing the Allium stents for two SRPF patients. The stent distorted and failed to dilate the stricture after self-expansion. This had not happened for the Resonance stent yet, suggesting the Resonance had more intensity against foreign force and were more suitable in severe ischemic fibrosis stenosis conditions secondary to radiation.

During follow-up, the Allium group showed 4 cases of displacement, accounting for 26% of the group cases. Among them, 2 cases were SRPF, 1 case was IRPF, and 1 case was post-lithotripsy stenosis. 2 of 4 patients received replacement of the thicker Allium stents; 1 patient received the Resonance stent replacement; 1 patient received observation. The rate was higher than previous literature, where 7 out of 49 (14.2%) stents migrated [18]. Meanwhile, only 1 patient with the Resonance stent had migration, but not necessary for intervention.

Stent occlusion was not rare for both the two types of stents. Moskovitz el at reported 1 occlusion case out of 49 Allium stent placements. Lopez et al. showed a high success rate of the Resonance stent in treating benign ureteral obstruction, with only 1 failed case because of the extremely tortuous ureter. Wang et al. reported a higher re-obstruction rate (22.7%) of the Resonance stent, but the failure mainly happened in the malignant cases with radiotherapy history. In our study, the Allium group had significant higher rate of postoperative re-obstruction, mostly in SRPF cases. Thus, close follow-up is necessary to discover the position changes or occlusion especially after the Allium stent placement. And the Resonance stents might be a safer choice for SRPF secondary to radiotherapy. During follow-up of RPF cases, images like plain X-ray help to understand whether the ureters and the stents are shifted to the midline and to decide whether to replace or remove the stents.

Irritable lower urinary tract symptoms are common complications of indwelling catheters. Likewise, moderate to severe OAB happened frequently in the Resonance group but not the Allium group. Some patients even requested to remove the Resonance stent due to disturbing OAB. Also, patients with the Resonance stents had significantly higher chance of recurrent urinary infection. On the contrary, the Allium stents were well tolerated. Those patients who had the Resonance stent withdrawn and replaced by the Allium stent were also satisfied with the relief of irritable symptoms. The Resonance stent is a full ureteral stent which is connected from the renal pelvis to the bladder, which may impair the peristaltic function of the ureter and leaves a “foreign body” inside the bladder. The Allium stent is segmental and only expands the narrowed ureter without affecting the peristalsis of other segments of the ureter. These reasons might contribute to the higher frequency of irritation and infection in the Resonance group. Although more patients with the Resonance stents had encrustation formed on the bladder and lower ureteral stents, the blood creatinine, GFR and CT images showed no signs of aggravated obstruction.

Some researcher proposed that metal stents are not suitable for benign ureteral obstruction, especially when ureteral stricture was caused by proliferation into the ureteral lumen. Careful selection of suitable cases before surgery and close follow-up after surgery are critical to the success of metal stent implantation [7]. In our study, there were 4 patients with refractory stenosis after ureterolithiasis surgery. The problem was completely solved by inserting Allium stent because the structure of the stent is a metal stent with double layers of polymer attached to the inside and outside of the metal mesh stent. The hydrophilic walls prevent the problem of the proliferation into the ureteral lumen. Also, according to our early experience, we placed tandem ureteral stents (TUS) in 3 patients with RPF but all stents failed at 3-month follow-up. Although studies showed TUS had good efficacy in benign conditions like ureteral stricture or severe stone disease [19], their efficacy in treating refractory cases with RPF needs further study.

In summary, both the Resonance and Allium stents could effectively protect the renal function. The Resonance stents showed higher frequency of irritable symptoms, while the Allium stents had more possibility of re-obstruction. The main limitation of the study is it small samples, mostly due to the rarity of non-malignant ureterostenosis like RPF. To evaluate the quality of life with ureteral stents, Ureteral Stent Symptom Questionnaire should be the most proper method. But we did not apply this questionnaire as it was not valid in the Chinese language at the early study period. Also, because the Resonance stents were introduced much earlier in our hospital, the follow-up time of the Resonance group was significantly longer than the Allium group. This bought bias to our study as the long-time efficacy and safety of the Allium stents were not possible to evaluate. For the same reason, more patients with IRPF were treated with the Resonance stents especially at the early stage of the study period. This bought bias to the disease consistency at baseline. The long-time efficacy and safely of the Allium stents to treat non-malignant ureterostenosis needs further study.

## Conclusion

Both the Resonance metallic stent and Allium stent can be applied to treat non-malignant refractory ureterostenosis with good efficacy and safety. However, the Resonance stent causes more irritative complications, and the Allium stent has higher possibility of re-obstruction. The efficacy and safety of the Allium stent still requires long term close follow-up.

## Supplementary Information

**Additional file 1:** The original clinical data including the patient demographics, the preoperative and/or postoperative parameters.

## Data Availability

Anonymous clinical data available in the supplementary material (see Additional file 1).

## Abbreviations

IRPF: Idiopathic retroperitoneal fibrosis
SRPF: Secondary retroperitoneal fibrosis
CT: Computed tomography
GFR: Glomerular filtration rate
OAB: Overactive bladder
OABSS: Overactive bladder symptom score
TUS: Tandem ureteral stents

## References

[1] Christman MS, L’esperance JO, Choe CH, Stroup SP, Auge BK (2009). Analysis of ureteral stent compression force and its role in malignant obstruction. J Urol.

[2] Blaschko SD, Deane LA, Alfred Krebs BSF (2007). In-vivo evaluation of flow characteristics of novel metal ureteral stent. J Endourol.

[3] Christman MS, L’esperance JO, Choe CH (2009). Analysis of ureteral stent compression force and its role in malignant obstruction. J Urol.

[4] Oderda M, Lacquaniti S, Fasolis G (2018). Allium stent for the treatment of a malignant ureteral stenosis: a paradigmatic case. Urologia.

[5] Duchene DA, Winfield HN, Cadeddu JA, Clayman RV, Gomella LG, Kavoussi LR, Mikhail AA, Park S, Permpongkosol S, Shalhav AL (2007). Multi-institutional survey of laparoscopic ureterolysis for retroperitoneal fibrosis. Urology.

[6] Stifelman MD, Shah O, Mufarrij P, Lipkin M (2008). Minimally invasive management of retroperitoneal fibrosis. Urology.

[7] Liatsikos E, Kallidonis P, Kyriazis I (2010). Ureteral obstruction: is the full metallic double-pigtail stent the way to go?. Eur Urol.

[8] Ito Y, Kikuchi E, Tanaka N, Miyajima A, Mikami S, Jinzaki M, Oya M (2011). Preoperative hydronephrosis grade independently predicts worse pathological outcomes in patients undergoing nephroureterectomy for upper tract urothelial carcinoma. J Urol.

[9] Vaglio A, Salvarani C, Buzio C (2006). Retroperitoneal fibrosis. Lancet.

[10] Vaglio A, Maritati F (2016). Idiopathic retroperitoneal fibrosis. J Am Soc Nephrol.

[11] Oshiro H, Ebihara Y, Serizawa H (2005). Idiopathic retroperitoneal fibrosis associated with immunohematological abnormalities. Am J Med.

[12] Neild GH, Rodriguez-Justo M, Wall C, Connolly JO (2006). Hyper-IgG4 disease: report and characterisation of a new disease. BMC Med.

[13] Fernando A, Pattison J, Horsfield C, D'Cruz D, Cook G, O'Brien T (2017). [^18^F]-fluorodeoxyglucose positron emission tomography in the diagnosis, treatment stratification, and monitoring of patients with retroperitoneal fibrosis: a prospective clinical study. Eur Urol.

[14] Benson AD, Taylor ER, Schwartz BF (2011). Metal ureteral stent for benign and malignant ureteral obstruction. J Urol.

[15] Polcari AJ, Hugen CM, López-Huertas HL (2010). Cost analysis and clinical applicability of the resonance metallic ureteral stent. Expert Rev Pharmacoecon Outcomes Res.

[16] López-Huertas HL, Polcari AJ, Acosta-Miranda A (2010). Metallic ureteral stents: a cost-effective method of managing benign upper tract obstruction. J Endourol.

[17] Wang HJ, Lee TY, Luo HL (2011). Application of resonance metallic stents for ureteral obstruction. BJU Int.

[18] Moskovitz B, Halachmi S, Nativ O (2012). A new self-expanding, large-caliber ureteral stent: results of a multicenter experience. J Endourol.

[19] Elsamra SE, Motato H, Moreira DM (2013). Tandem ureteral stents for the decompression of malignant and benign obstructive uropathy. J Endourol.

