# Supplementary material for: The Resonance and the Allium ureteral stents in the treatment of non-malignant refractory ureterostenosis
Source: BMC Urol. 2021 Apr 7;21:53. doi: 10.1186/s12894-021-00815-6 (PMC8025479; doi:10.1186/s12894-021-00815-6)
Supplement: Supplementary file 1 — Additional file 1: The original clinical data including the patient demographics, the preoperative and/or postoperative parameters. [file 12894_2021_815_MOESM1_ESM.docx]

**The Resonance and the Allium ureteral stents in the treatment of non-malignant refractory ureterostenosis**

Wei Gao^1^, Tianying Xing^1^, Tongwen Ou^1^

1. Department of Urology, Xuanwu Hospital, Capital Medical University, Beijing 100053, China

Corresponding to Tongwen Ou

Telephone: 0086-1083198388

Email: [outongwen@sohu.com](mailto:outongwen@sohu.com)

Table 1： Patients information The Resonance stent group

| No. | Gender | Age | Follow-up (months) | Primary disease |
| --- | --- | --- | --- | --- |
| 1 | M | 53 | 84 | IRPF |
| 2 | F | 55 | 47 | IRPF |
| 3 | M | 64 | 28 | IRPF |
| 4 | M | 74 | 37 | IRPF |
| 5 | M | 64 | 75 | IRPF |
| 6 | M | 63 | 75 | IRPF |
| 7 | M | 61 | 26 | IRPF |
| 8 | M | 44 | 36 | IRPF |
| 9 | M | 71 | 63 | IRPF |
| 10 | M | 54 | 23 | IRPF |
| 11 | F | 54 | 33 | IRPF |
| 12 | M | 66 | 18 | IRPF |
| 13 | F | 63 | 17 | IRPF |
| 14 | M | 16 | 2 | IRPF |
| 15 | F | 47 | 23 | SRPF |
| 16 | M | 39 | 10 | SRPF |
| 17 | F | 60 | 13 | SRPF |
| 18 | F | 54 | 42 | SRPF |

IRPF: Idiopathic retroperitoneal fibrosis; SRPF: Secondary retroperitoneal fibrosis.

Table 2: Patients information The Allium stent group

| No. | Gender | | Age | Follow-up (months) | Primary disease |
| --- | --- | --- | --- | --- | --- |
| 1 | M | 71 | | 2 | SRPF |
| 2 | M | 46 | | 15 | Ureteral lithotripsy |
| 3 | F | 24 | | 15 | IRPF |
| 4 | F | 50 | | 15 | SRPF |
| 5 | F | 62 | | 14 | IRPF |
| 6 | M | 68 | | 13 | IRPF |
| 7 | M | 40 | | 12 | SRPF |
| 8 | M | 61 | | 12 | IRPF |
| 9 | M | 50 | | 11 | Ureteral lithotripsy |
| 10 | F | 61 | | 10 | SRPF |
| 11 | F | 54 | | 9 | SRPF |
| 12 | M | 61 | | 4 | IRPF |
| 13 | M | 51 | | 4 | Ureteral lithotripsy |
| 14 | F | 46 | | 4 | SRPF |
| 15 | M | 37 | | 1 | Ureteral lithotripsy |

IRPF: Idiopathic retroperitoneal fibrosis; SRPF: Secondary retroperitoneal fibrosis.

Table 3: Preoperative and postoperative examinations of the patients with Resonance stents

| No. | Gender | Age | Pr Cr | Po Cr | Pr G L | Pr G R | Pr R F L | Pr R F R | Po G L | Po G R | Po R F L | Po R F R | Pr HN | Po HN |
| --- | --- | --- | --- | --- | --- | --- | --- | --- | --- | --- | --- | --- | --- | --- |
| 1 | M | 53 | 83 | 65 | 27.4 | 42.21 | 39.36 | 60.64 | 26.59 | 47.73 | 35.78 | 64.22 | 3 | 2 |
| 2 | F | 55 | 95 | 75 | 8.56 | 50.08 | 14.6 | 85.4 | 9.71 | 40.92 | 19.18 | 80.82 | 3 | 2 |
| 3 | M | 64 | 135 | 108 | 11.1 | 18.9 | 37.1 | 62.9 | NA | NA | NA | NA | 2 | 2 |
| 4 | M | 74 | 146 | NA | 1.405 | 46.3 | 2.9 | 97.1 | NA | NA | NA | NA | 2 | 2 |
| 5 | M | 64 | 135 | 104 | 49.5 | 11.2 | 81.5 | 18.5 | 50.43 | 10.69 | 82.5 | 17.5 | 4 | 4 |
| 6 | M | 63 | 67 | 65 | 43.2 | 47.6 | 47.6 | 52.4 | 49.41 | 50.06 | 49.7 | 50.3 | 2 | 1 |
| 7 | M | 61 | 523 | 153 | 11.2 | 25.7 | 30.4 | 69.6 | 13.4 | 32.5 | 29.2 | 70.8 | 3 | 2 |
| 8 | M | 44 | 266 | 103 | 3.83 | 37.35 | 9.3 | 90.7 | 1.85 | 41.37 | 4.3 | 95.7 | 2 | 0 |
| 9 | M | 71 | 95 | 83 | 9.56 | 44.7 | 17.6 | 82.4 | 10.46 | 44.6 | 19.0 | 81.0 | 4 | 4 |
| 10 | M | 54 | 81 | 84 | 34.83 | 49.2 | 41.4 | 58.6 | NA | NA | NA | NA | 3 | 2 |
| 11 | F | 54 | 752 | 94 | 8 | 24.56 | 24.57 | 75.43 | 3.9 | 32.88 | 10.6 | 89.4 | 3 | 2 |
| 12 | M | 66 | 78 | 93 | 28.62 | 37.24 | 43.46 | 56.44 | 33.64 | 50.4 | 40.03 | 59.97 | 2 | 2 |
| 13 | F | 63 | 72 | 70 | NA | NA | NA | NA | NA | NA | NA | NA | 4 | 2 |
| 14 | M | 16 | 958 | 109 | NA | NA | NA | NA | NA | NA | NA | NA | 3 | 1 |
| 15 | F | 47 | 71 | 62 | 35.69 | 12.98 | 73.33 | 26.67 | 41.39 | 41.58 | 49.89 | 50.11 | 4 | 4 |
| 16 | M | 39 | 77 | 83 | 40.27 | 38.4 | 51.19 | 48.81 | 53.26 | 38.97 | 57.75 | 42.25 | 3 | 3 |
| 17 | F | 60 | 70 | 95 | 36.85 | 25.37 | 59.23 | 40.77 | 29.7 | 17.12 | 63.43 | 36.57 | 2 | 2 |
| 18 | F | 54 | 75 | 81 | 19.78 | 60.52 | 24.63 | 75.37 | 17.09 | 49.45 | 25.68 | 74.32 | 3 | 2 |

Pr Cr: Preoperative Creatinine; Po Cr: Postoperative creatinine; Pr G L: Preoperative GFR Left (ml/min); Pr G R: Preoperative GFR right ml/min; Pr R F L: Preoperative Renal Function proportion Left (%): Pr R F R: Preoperative Renal function proportion Right (%); Po G L: Postoperative GFR Left kidney (ml/min); Po G R: Postoperative GFR Right (ml/min); Po R F L:;Postoperative Renal Function proportion Left (%); Po R F R: Postoperative Renal Function proportion Right (%); Pr HN: Preoperative hydronephrosis grade; Po HN: Postoperative hydronephrosis grade.

Table 4: Preoperative and postoperative examinations of the patients with Allium metal stents

| No. | Gender | Age | Pr Cr | Po Cr | Pr G L | Pr G R | Pr R F L% | Pr R F R% | Po G L | Po G R | Po R F L% | Po R F R% | Pr HN | Po HN |
| --- | --- | --- | --- | --- | --- | --- | --- | --- | --- | --- | --- | --- | --- | --- |
| 1 | M | 53 | 78 | 103 | 29.18 | 37.7 | 43.63 | 56.37 | NA | NA | NA | NA | 2 | 2 |
| 2 | F | 55 | 73 | 62 | 70.39 | 68.63 | 50.63 | 49.37 | 65.6 | 63.4 | 50.85 | 49.15 | 2 | 1 |
| 3 | M | 64 | 145 | 156 | NA | NA | NA | NA | NA | NA | NA | NA | 3 | 1 |
| 4 | M | 74 | 71 | 62 | 41.39 | 41.58 | 49.89 | 50.11 | 39.35 | 40.6 | 49.22 | 50.78 | 4 | 2 |
| 5 | M | 64 | 75 | 83 | 9.71 | 40.92 | 19.18 | 80.82 | 10.04 | 45.23 | 18.17 | 81.83 | 2 | 1 |
| 6 | M | 63 | 93 | 100 | 33.64 | 50.4 | 40.03 | 59.97 | 28.32 | 41.97 | 40.29 | 59.71 | 2 | 1 |
| 7 | M | 61 | 87 | 93 | 53.26 | 38.97 | 57.75 | 42.25 | NA | NA | NA | NA | 3 | 2 |
| 8 | M | 44 | 265 | 85 | 31.69 | 26.54 | 54.42 | 45.58 | 43.22 | 47.51 | 47.64 | 52.36 | 2 | 1 |
| 9 | M | 71 | 64 | 67 | 26.25 | 42.18 | 38.36 | 61.64 | 21.45 | 48.39 | 30.71 | 69.29 | 4 | 3 |
| 10 | M | 54 | 84 | 71 | 29.7 | 17.12 | 63.43 | 36.57 | NA | NA | NA | NA | 2 | 1 |
| 11 | F | 54 | 86 | 85 | 12.91 | 43.24 | 22.99 | 77.01 | 17.09 | 49.45 | 25.68 | 74.32 | 4 | 4 |
| 12 | M | 66 | 70 | 68 | 26.59 | 47.73 | 35.78 | 64.22 | 25.93 | 40.96 | 38.77 | 61.23 | 2 | 1 |
| 13 | F | 63 | 103 | 85 | 48.53 | 19.29 | 71.56 | 28.44 | 62.07 | 17.61 | 77.9 | 22.1 | 4 | 4 |
| 14 | M | 16 | 35 | 35 | NA | NA | NA | NA | NA | NA | NA | NA | 1 | 0 |
| 15 | F | 47 | 115 | 110 | 20.67 | 42.52 | 32.71 | 67.29 | NA | NA | NA | NA | 4 | 3 |

Pr Cr: Preoperative Creatinine; Po Cr: Postoperative creatinine; Pr G L: Preoperative GFR Left (ml/min); Pr G R: Preoperative GFR right ml/min; Pr R F L: Preoperative Renal Function proportion Left (%): Pr R F R: Preoperative Renal function proportion Right (%); Po G L: Postoperative GFR Left kidney (ml/min); Po G R: Postoperative GFR Right (ml/min); Po R F L:;Postoperative Renal Function proportion Left (%); Po R F R: Postoperative Renal Function proportion Right (%); Pr HN: Preoperative hydronephrosis grade; Po HN: Postoperative hydronephrosis grade.

Table 5: Postoperative safety parameters for the Patients with the Resonance Stents

| No. | Gender | Age | OABSS | RI(Y:1N:2) | Pain(Y:1N:2) | Move(Y:1N:2) | Encrustation(Y:1N:2) | Obstruction(Y:1N:2) |
| --- | --- | --- | --- | --- | --- | --- | --- | --- |
| 1 | M | 53 | 2 | 2 | 2 | 2 | 1 | 2 |
| 2 | F | 55 | 12 | 1 | 1 | 2 | 1 | 2 |
| 3 | M | 64 | 2 | 2 | 2 | 2 | 2 | 2 |
| 4 | M | 74 | 8 | 1 | 1 | 2 | 2 | 2 |
| 5 | M | 64 | 2 | 2 | 2 | 2 | 1 | 2 |
| 6 | M | 63 | 13 | 1 | 1 | 1 | 2 | 2 |
| 7 | M | 61 | 3 | 2 | 2 | 2 | 2 | 2 |
| 8 | M | 44 | 2 | 2 | 2 | 2 | 1 | 2 |
| 9 | M | 71 | 2 | 2 | 2 | 2 | 1 | 2 |
| 10 | M | 54 | 3 | 2 | 2 | 2 | 1 | 2 |
| 11 | F | 54 | 1 | 2 | 2 | 2 | 1 | 2 |
| 12 | M | 66 | 12 | 1 | 1 | 2 | 1 | 2 |
| 13 | F | 63 | 1 | 2 | 2 | 2 | 2 | 2 |
| 14 | M | 16 | 6 | 2 | 1 | 2 | 2 | 2 |
| 15 | F | 47 | 9 | 1 | 1 | 2 | 1 | 2 |
| 16 | M | 39 | 5 | 1 | 2 | 2 | 1 | 2 |
| 17 | F | 60 | 5 | 2 | 2 | 2 | 2 | 2 |
| 18 | F | 54 | 8 | 1 | 1 | 2 | 1 | 2 |

OABSS: Overactive Bladder Symptom Score; RI: Refractory Infection; Y: Yes; N: No.

Table 6: Postoperative safety parameters for the Patients with the Allium Stents

| No. | Gender | Age | OABSS | RI(Y:1N:2) | Pain(Y:1N:2) | Move(Y:1N:2) | Encrustation(Y:1N:2) | Obstruction(Y:1N:2) |
| --- | --- | --- | --- | --- | --- | --- | --- | --- |
| 1 | M | 71 | 1 | 2 | 2 | 1 | 2 | 1 |
| 2 | M | 46 | 2 | 2 | 2 | 2 | 2 | 2 |
| 3 | F | 24 | 4 | 2 | 2 | 2 | 2 | 2 |
| 4 | F | 50 | 3 | 2 | 2 | 2 | 2 | 2 |
| 5 | F | 62 | 2 | 2 | 2 | 2 | 2 | 2 |
| 6 | M | 68 | 2 | 2 | 2 | 2 | 2 | 2 |
| 7 | M | 40 | 1 | 2 | 2 | 2 | 2 | 2 |
| 8 | M | 61 | 3 | 2 | 2 | 1 | 2 | 1 |
| 9 | M | 53 | 3 | 2 | 2 | 1 | 2 | 1 |
| 10 | F | 61 | 1 | 2 | 2 | 2 | 2 | 2 |
| 11 | F | 56 | 1 | 1 | 1 | 1 | 2 | 1 |
| 12 | M | 61 | 2 | 2 | 2 | 2 | 2 | 2 |
| 13 | M | 51 | 1 | 2 | 2 | 2 | 1 | 1 |
| 14 | F | 46 | 5 | 2 | 2 | 2 | 2 | 2 |
| 15 | M | 37 | 0 | 2 | 2 | 2 | 2 | 2 |

OABSS: Overactive Bladder Symptom Score; I: Refractory Infection; Y: Yes; N: No.
